# Variant identification and genotyping strategy for the *smg-1(r861)* allele in *Caenorhabditis elegans*

**DOI:** 10.17912/micropub.biology.002026

**Published:** 2026-02-05

**Authors:** Michael Zoberman, John A. Calarco

**Affiliations:** 1 Cell and Systems Biology, University of Toronto, 25 Harbord Street, Toronto, Ontario, Canada, M5S 3G5

## Abstract

The *C. elegans* *smg-1* gene encodes a PI3K-related kinase responsible for initiating the process of nonsense-mediated decay (NMD). The *smg-1 ( r861 )* allele is a strong loss-of-function variant that disrupts NMD activity leading to the stabilization of transcripts containing premature stop codons (PTCs). This allele has been used extensively in studies of RNA surveillance, transcript stability, and transgene regulation. Despite its widespread use, the mutation in *smg-1 ( r861 )* has not been reported, and identification is often based solely on phenotype. Here, we identify the underlying mutation and present a restriction digestion-based genotyping strategy that enables quick confirmation of the *smg-1 ( r861 )* allele.

**Figure 1. Identification of smg-1(r861) variant sequence:**
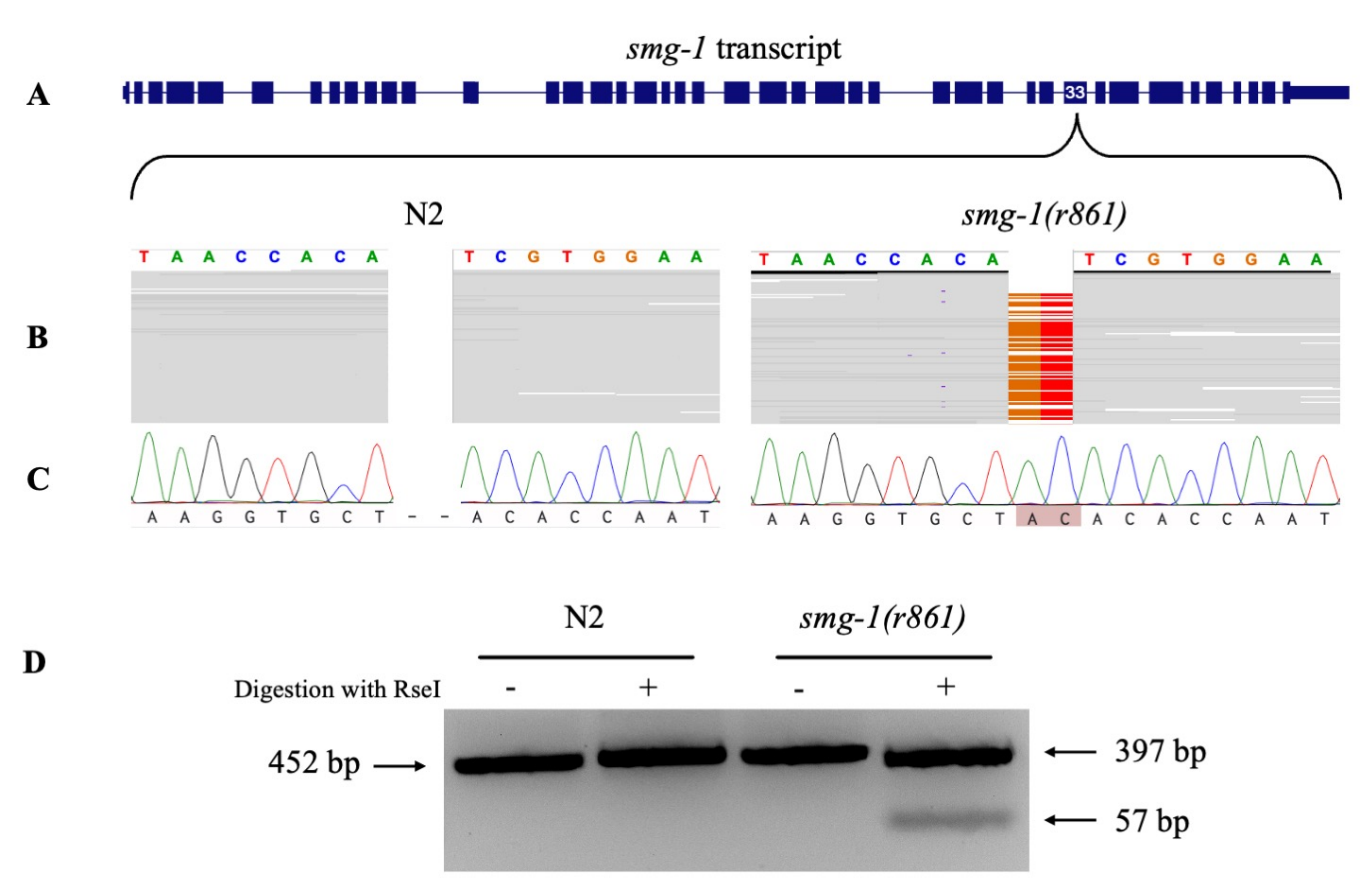
**A)** Exon architecture of the *smg-1* gene with exon 33 highlighted as the mutation site in the *smg-1 ( r861 )* allele. Image was obtained from the UCSC Genome Browser (Perez et al., 2025). **B)** Alignment of RNA-seq data from N2 and *smg-1 ( r861 )* highlighting a two-base insertion within the smg-1 gene in the mutant. **C)** Chromatograms representing Sanger sequencing of amplicons from the *smg-1* gene in N2 and *smg-1 ( r861 )* . **D)** Agarose gel displaying fragment sizes of the *smg-1* amplicon from N2 and *smg-1 ( r861 )* . Treatment of the amplicon from *smg-1 ( r861 )* with RseI causes fragmentation while the amplicon from N2 is insensitive to RseI.

## Description

Nonsense-mediated decay (NMD) is an evolutionarily conserved RNA surveillance system that degrades transcripts containing premature termination codons (Kurosaki et al., 2019). *C. elegans* SMG-1 , an ortholog of human SMG1, is a PI3K-related kinase and a key component of the NMD pathway. The role of SMG-1 in NMD has been well characterized; it phosphorylates the SMG-2 /UPF1 RNA helicase to promote degradation of PTC-containing transcripts (Grimson et al., 2004; Johns et al., 2007; Page et al., 1999). SMG-1 also functions in additional pathways, including DNA double-stranded break repair (González-Huici et al., 2017; Kamp et al., 2022) and regulation of lifespan through DAF-16 , the *C. elegans* ortholog of mammalian FOXO transcription factors (Masse et al., 2008).

The *smg-1 ( r861 )* allele was originally identified in a forward genetic screen by Hodgkin et al. (1989) as a suppressor of an *unc-54* nonsense allele. *smg-1 ( r861 )* is a strong loss-of-function allele that is defective in NMD, allowing transcripts containing PTCs to escape degradation. Despite its frequent use in studies of RNA surveillance and transgene expression, to our knowledge, the precise mutation in *smg-1 ( r861 )* has not previously been reported.

To identify the underlying mutation, we analyzed a publicly available RNA sequencing dataset containing both wild-type (Bristol N2 ) and *smg-1 ( r861 )* strains (Rubio-Peña et al., 2015). Alignment of the *smg-1* transcripts revealed a two-base pair insertion in exon 33 of the gene in *smg-1 ( r861 )* animals that was absent in the wild-type ( Figure 1A, B). This insertion lies within the conserved PI3K/PI4K catalytic domain at residue P1846 and introduces a frameshift that results in a premature termination codon (PTC) shortly downstream of the insertion.

Interestingly, the *smg-1* transcripts remain detectable in *smg-1 ( r861 )* animals, consistent with a failure to trigger NMD. It is therefore likely that SMG-1 kinase function is required for degradation of its own PTC-containing transcript in *smg-1 ( r861 )* mutants. However, a previous study found that SMG-1 protein is undetectable by Western blot in *smg-1 ( r861 )* animals using an antibody directed against an epitope upstream of the PTC (Grimson et al., 2004), suggesting that the truncated transcript is either not efficiently translated or the resulting protein is rapidly degraded.

To validate the identified mutation, we amplified a 452 bp region of genomic DNA from the *smg-1* gene surrounding the predicted insertion site in wild-type and *smg-1 ( r861 )* animals. Sanger sequencing of the PCR products confirmed the presence of an AC dinucleotide insertion at Chromosome I:6,903,692 in *smg-1 ( r861 )* , which was absent in wild-type ( Figure 1C ).

Notably, this insertion creates a unique RseI restriction site that is not present in the wild-type sequence. To develop a simple genotyping assay, we digested the 452 bp PCR product with RseI. Digestion of the *smg-1 ( r861 )* amplicon produced two fragments of 397 bp and 57 bp, while the wild-type amplicon remained undigested. This approach therefore provides a rapid and reliable method for identifying the *smg-1 ( r861 )* allele based on restriction fragment length polymorphism ( Figure 1D ). We anticipate that our assay will be useful for future experiments involving crossing the *r861* allele into other relevant genetic backgrounds.

## Methods

RNA seq analysis

SRA files from the GEO dataset GSE72952 were aligned to the WBcel235 reference genome using STAR (Dobin et al., 2013).

Worm maintenance

*C. elegans* strains were maintained under standard conditions as described by Brenner (1974).

The following strains were used: Bristol N2 and TR1331 *smg-1 ( r861 )* I.

All strains were provided by the CGC, which is funded by the NIH Office of Research Infrastructure Programs (P40 OD010440).

Amplification, sequencing, and digestion

Genomic DNA was extracted using a protocol based on Williams et al. (1992). Briefly, a worm lysis buffer was prepared with 50 mM Tris-HCl pH 8.0, 50 mM KCl, 2.5 mM MgCl _2_ , 0.45% NP-40, 0.45% Tween 20, and 68 μg/mL proteinase K. Single adult worms were added to 6 μl lysis buffer and incubated at 65 °C for 1 hr 5 min, then 95 °C for 15 min.

Amplicons were generated with Vazyme Rapid Taq (Cat. no. P222) using 1 μl of lysed worm solution with the forward primer 5′-GTTATTCCACTTGGACCACG-3′ and reverse primer 5′-CATCCAAAGCTCACGACTG-3′. PCR was performed with an annealing temperature of 55.7 °C, 10 seconds extension, and 35 cycles. Amplicons were cleaned with Zymo Research DNA Clean & Concentrator (Cat. no. D4014).

Sanger sequencing of cleaned amplicons was performed by The Centre for Applied Genomics, The Hospital for Sick Children, Toronto, Canada.

Amplicons were digested using ThermoFisher RseI (Cat. no. ER2001) by incubating for 3 hrs at 37 °C. A 2% agarose gel in 1x TAE was run at 120 V for 60 min with the digested DNA. The gel was imaged after 500 ms UV light exposure using a Bio-Rad Gel Doc XR+ imaging system (Universal Hood II) and Image Lab software (version 6.0.1).
